# Molecular Therapy Advances: Building the bridge between discovery and cure

**DOI:** 10.1016/j.omtm.2025.101641

**Published:** 2025-11-17

**Authors:** Mohamed Abou-el-Enein

**Affiliations:** Editor-in-Chief, Molecular Therapy Advances (formerly MTMCD), Executive Director, USC/CHLA Cell Therapy Program, Associate Professor of Clinical Medicine (Oncology), Pediatrics, and Stem Cell Biology & Regenerative Medicine, Keck School of Medicine of USC, University of Southern California

## Main text

If I have seen further, it is by standing on the shoulders of giants.—Isaac Newton

When I first proposed renaming Molecular Therapy Methods & Clinical Development to Molecular Therapy Advances, it was out of conviction that our journal must evolve hand in hand with the most daring breakthroughs in gene and cell therapy. This rebranding represents a deeper commitment to publish science that shapes the future of medicine. For many years, “personalized therapies” was a hopeful phrase. Today, it describes real medicines, real cures, and real transformations in patients’ lives. Our role is to share these stories and show how each discovery moves us closer to a cure.

The last decade brought progress that few would have predicted. The 2020 Nobel Prize in Chemistry awarded to Jennifer Doudna and Emmanuelle Charpentier recognized the transformation of CRISPR-Cas9 into a programmable genome editing system.^1^ That discovery validated gene editing as a foundation of modern biology. Just 4 years later, the 2024 Nobel Prize in Chemistry honored David Baker, Demis Hassabis, and John Jumper for advances in computational protein design and structure prediction.^2^^,^^3^ Their pioneering work is reshaping how we model, engineer, and optimize proteins for therapeutic use.

In 2025, the Nobel Prize in Physiology or Medicine recognized Mary Brunkow, Fred Ramsdell, and Shimon Sakaguchi for their discoveries regarding regulatory T cells and peripheral immune tolerance.^4^^,^^5^ Regulatory T cells are already being developed as therapeutic agents to restore immune balance in autoimmune and inflammatory diseases.^6^ Equally deserving of recognition are advances in chimeric antigen receptor (CAR) T cell therapies, which have transformed cancer treatment and are now expanding into new disease areas.^7^ The same “giants” who built these foundations continue to propel the field forward, supported by a global effort committed to turning discovery into therapy.

At the same time, converging technologies are reshaping what is possible in laboratories and clinical trials. *In vivo* CAR T therapy is progressing from concept to clinical reality, using targeted delivery systems to reprogram immune cells directly within the body.^8^ Machine learning is accelerating the design of adeno-associated virus capsids with greater tissue specificity and reduced immunogenicity.^9^ Base editing has already reached the clinic,^10^ offering correction of inherited pediatric diseases that were once beyond therapeutic reach. In parallel, advances in single-cell and multi-omic integration,^11^ together with high-dimensional spectral flow cytometry,^12^ now provide unprecedented resolution into cell behavior. Such systems-level insights are becoming essential for understanding therapeutic durability and refining product design.

This is the vision that brings Molecular Therapy Advances to life. Our goal is to capture the remarkable progress unfolding across the field. We feature studies that translate advances in gene and cell therapy technologies into meaningful patient outcomes. We also welcome manuscripts that confront unexpected findings or challenge prevailing models, since genuine progress often emerges from reexamining what we think we know. Methodological rigor, reproducibility, and transparency will remain at the core of what we publish.

The bridge between discovery and cure is not a metaphor. It is a collective endeavor that depends on collaboration among molecular biologists, immunologists, microbiologists, computational scientists, clinicians, engineers, and many other disciplines. Molecular Therapy Advances aims to serve as both the path and the meeting ground for that effort. Thank you for trusting this journal, this mission, and this shared journey. May your discoveries find a home here, and may we continue to cross this bridge together—bringing insight, compassion, and precision to the science of cure.
